# Specific Autobiographical Recall Mediates Impact of Cognition and Depression on Independence Function and Well-Being in Older Adults

**DOI:** 10.3389/fpsyg.2021.652600

**Published:** 2021-04-20

**Authors:** Carol A. Holland, Alexis Boukouvalas, Danielle Clarkesmith, Richard Cooke

**Affiliations:** ^1^Division of Health Research, Centre for Ageing Research, Lancaster University, Lancaster, United Kingdom; ^2^Molecular Sciences, University of Manchester, Manchester, United Kingdom; ^3^Aston Research Centre for Healthy Ageing, Aston University, Birmingham, United Kingdom; ^4^Department of Psychology, University of Liverpool, Liverpool, United Kingdom

**Keywords:** extra care supported living, autobiographical memory, cognitive functioning, independence, active ageing, mental well-being, depression

## Abstract

Autobiographical memory specificity has been associated with cognitive function, depression, and independence in older adults. This longitudinal study of 162 older adults moving to active supported living environments tracks changes in the role of the ability to recall specific autobiographical memory as a mediator between underlying cognitive function, or depression, and outcome perceived health or independence (e.g., Instrumental Activities of Daily Living, IADLs), across 18 months, as compared with controls not moving home. Clear improvements across time in autobiographical specificity were seen for residents but not controls, supporting the role of a socially active environment, and confirmed by correlation with number of activities reported in diaries, although the impact of diary activities on the effect of time on autobiographical specificity was not found. The role of autobiographical specificity in mediating general cognition and outcome functional limitations was clear for social limitations at 12 and 18 months, but its role in mediating effects of executive function and perceived health persisted throughout. The role of specificity in mediating between depression and perceived health, IADLs, and Functional Limitations persisted throughout. Analysis examining autobiographical specificity and depression as joint mediators between cognition and independence showed a forward effect such that higher specificity scores reduced the negative mediation effect of depression on independence. Finally, data showed the reduction of many of these mediations over time, supporting the role of autobiographical memory in times of change in a person's social situation. Data support potential autobiographical memory intervention development.

## Introduction

Evidence suggests that retirement villages have a positive impact on the health and well-being of residents (e.g., Biggs et al., 2000; Darton et al., 2011). For example, Jenkins et al. (2002) found that engaging in more activities in such active environments was associated with increased health-related quality of life. Previous analyses with the sample studied here (people aged 60 plus moving to retirement villages) indicated significant increase in social engagement and activity from moving in to 3 months later, and a positive impact of the transition phase on aspects of cognitive function, self-reported functional limitations, and mental well-being (Holland et al., 2015, 2017). In particular, the measure of autobiographical memory used, a measure of ability to be specific as opposed to over-general in recall (e.g., Williams and Broadbent, 1986), was predictive of changes in functional limitations, particularly with regard to communication limitations. In further analyses over a longer period, autobiographical memory specificity (AMS) showed an overall positive effect of time (improving by 24%, on average) whereas a control group who had not moved to the retirement villages showed a decline (Holland et al., 2019). In these data, we also found that the measure of autobiographical memory used contributed significantly to the prediction of loneliness.

Well-established research links ability to recall autobiographical events that are specific in time and place to depression (e.g., Williams et al., 2007) and to important social functions related to maintaining and developing relationships (Alea and Bluck, 2003). Over-general autobiographical retrieval has been associated with impaired social problem solving (Goddard et al., 1997), including in older participants (Beaman et al., 2007; Leahy et al., 2018a,b), and greater negative emotional response to traumatic life events (Raes et al., 2006). AMS also associates with cognitive functions, specifically executive function in depression (Dalgleish et al., 2007) and in healthy older age (Piolino et al., 2010; Holland et al., 2012). Ability to be specific in autobiographical recall also reduces with increasing age (Holland and Rabbitt, 1990; Piolino et al., 2002, 2010; Ros et al., 2010; Holland et al., 2012), with Holland et al. (2012) confirming that this is a robust finding even controlling for depression.

Given the functional role of autobiographical memory, and its socio-emotional nature (links to emotional health and personal relationships), these associations are important. They link basic cognitive indices with the functional memory and emotional well-being that affect everyday social engagement and the problem solving involved in coping, perhaps compensating for other impairments in an ongoing fashion, and maintaining independent function and quality of life. Additionally, given the impact of depression on cognitive performance and its implication as a risk factor for dementia (Jorm, 2001; da Silva et al., 2013), identifying the role of a function that has an impact on depression or effects of depression is crucial. Thus, it can be proposed that autobiographical memory function may mediate the impacts of cognitive function and depressive states on outcome functional independence and communication for older adults. This is particularly important given that this function, AMS, has been shown to be amenable to rehabilitation in people with depression (Raes et al., 2009) and in healthy and frailer older people (Leontjevas et al., 2013; Leahy et al., 2018b).

In this study we therefore aim to examine the impact of moving to a supported active environment on AMS over the ensuing 18 months, in a group of older adults with varying function and health, in relation to moving to the new environment and also specifically in relation to activity in which people actually engage. We take previous analyses further by examining the role of AMS in relationships with well-being and independence indices over the first 18 months of living in the active environment, and its association with numbers of activities engaged in.

We also aim to examine the role of AMS in mediating effects of depression or underlying cognitive function on outcome independence and functional limitations, and perceived well-being. Improvement in such outcomes has significant implications for quality of life and need for care, and demonstrating such impacts in this context validates the role of supported, socially accessible environments. Establishing understanding of how autobiographical memory has its impact on depression and independence function in older adults is both theoretically important and vital to inform future interventions.

In summary, hypotheses are:

1. Moving to an active supported living environment will influence AMS, longitudinally and compared to a control sample.
2. AMS will be associated with number of activities engaged in.
3. AMS acts as an intermediary functional measure of memory between basic cognitive function in older age and perceived health, social functioning and independence outcomes.
4. AMS acts as an intermediary functional measure of memory between underlying mood or depression in older age and perceived health, social functioning and independence outcomes.
5. Given previous evidence of a relationship between AMS and depressive symptoms, AMS will have an impact on the mediating effect of depression between underlying cognitive functioning and outcome independence limitations.
6. Any improvement in AMS would have a longitudinal influence on functional limitations and independence indicators.

## Method

### Participants

This study assessed 162 new residents moving into 13 different “extra care^1^” retirement villages and schemes, in comparison to a control group of 39 older adults who remained in their original homes; mean ages (SD) 75.15 years (8.16) and 71.81 years (6.30), respectively. Controls were from a volunteer panel administered by the university, living in similar areas of the UK Midlands from which residents originated. The age difference is statistically significant, *F*_(1,192)_ = 4.58, *p* < 0.05 and so age is added as a covariate in analyses on AMS, given AMS correlates with age. The inclusion criteria were age ≥60 years and with mental capacity to understand participant information and consent. Consent was re-confirmed at each appointment. 17% percent of new residents took part.

Assessments were repeated at 3, 12, and 15 or 18 months after moving in. In order to account for anticipated attrition, new participants were added at each time point who had been living in the establishments for the correct duration (e.g., at the 3 month follow up, people who had lived in the extra care environments for 3 months but not originally volunteered were added, etc.). This recommended strategy contributes towards controlling for uneven attrition and practice effects (Hofer and Sliwinski, 2006). This means that time is treated as a variable in analyses, as opposed to repeated measures comparisons being conducted. This strategy and attrition data are detailed in Appendix 1 in Supplementary Material. The overall attrition rate was 38.3% over 18 months. There were no significant differences between those who joined later and original participants in any of the measures.

### Measures

- Cognitive function was assessed using the Addenbrooke's cognitive examination III (ACE-R^2^) (Mioshi et al., 2006). Maximum score is 100, with <88 indicating significant impairment (Noone, 2015). The fluency sub-scale (maximum score 14) was used to indicate executive function.
- Autobiographical memory was assessed using a modified version (Dalgleish et al., 2007) of the autobiographical memory test (AMT) (Williams and Broadbent, 1986). Participants were asked to retrieve different specific autobiographical memories in response to 10 cue words (five emotionally positive, five negative, e.g., clumsy, clever, nervous, safe). Cue words were adapted from Williams and Broadbent (1986), also impacted by feedback from many older participants on words used in previous studies, and by previous analyses of words that really did engender positive and negative memories (Holland et al., 2012). Examples were given to ensure understanding (see Appendix 2 in Supplementary Material for instructions), and the test was recorded for later inter-rater reliability checking. A specific memory was defined as an event that occurred at a specific time and place and lasted less than a day. Number of specific memories recalled from the 10 cues is taken as the score. Inter-rater reliability with the different scorers was checked by the first author, as part of scorers' training, with excellent agreement found. Cue lists were used in a counterbalanced manner.
- The Hospital Anxiety and Depression Scale (HADS) (Zigmond and Snaith, 1983) depression component was used to measure depressive symptoms, or negative mood. This self-report measure can detect presence and severity of anxiety and depression. The sub-score for depression (0–21) was used in the current study, higher scores indicating more depressive symptoms.
- Instrumental activities of daily living (IADL, scores: 0–8, higher scores indicating good functional abilities) (Lawton and Brody, 1969) and Functional Limitations Profile (FLP, scores 0–1,150, higher scores indicating more functional limitations) (Pollard and Johnston, 2001) were used as indicators of functional independence. FLP sub-constructs investigated were Recreation, Social, and Communication limitations in addition to total FLP score.
- Participants rated their self-perceived health, converted to a numerical scale: 5 = excellent to 1 = poor, and treated as an interval variable (Holland et al., 2017) based on meta-analysis indication of a dose-response pattern such that probability of death is highest for the lowest category (Idler and Benyamini, 1997).
- A smaller group (57 residents, 22 control) kept 2-week activity diaries before each assessment. An example of the diary with instructions is given in Appendix 3 in Supplementary Material, along with example coding structure. Activities were categorised broadly into social, physical, and intellectual engagement.

### Procedure

New residents were given study information by their village well-being advisor and interested residents gave permission for researchers to telephone them to arrange a meeting, at which participants gave informed consent prior to completing assessments. Potential control participants were invited by letter or email, and those who volunteered were contacted, informed and consent taken in the same way. Ethical approval was given by the University Research Ethics Committee, Reference 565. The study is an observational cohort study, with no random assignment to resident or control group. Differences between groups are detailed in previous reports (Holland et al., 2015). Although they were matched in age range, controls were younger on average than residents (75.89 vs. 71.89 years, *F*_(1,192)_ = 4.58, *p* < 0.05) and had fewer health conditions (not-significant, *F*_(1,192)_ = 0.97, *p* > 0.05). Data were collected by university researchers with a background in psychology; some initial specific health items were collected by retirement village nurses (well-being advisors).

### Analysis Plan

To determine longitudinal effect (effect of time), latent growth curve models (e.g., Singer and Willett, 2003) were conducted for autobiographical memory test score (AMT) with group (residents vs. controls) and age as covariates (given the group difference in age). Diary data were added to determine whether recorded activity influenced longitudinal change in AMT. To determine association with diary activities, Pearson's correlation coefficients were computed.

Hypotheses regarding mediation were assessed using a bootstrapping approach. Finally, a multiple mediation model was used to examine the combined role of mediation of AMS and depression on outcome functional independence. The criterion for determining statistical significance was *p* = 0.05, and *N* = 10,000 bootstrap iterations were used to estimate confidence intervals. The bootstrap approach used was that of Preacher and Hayes (2008), with code used based on Type 1, BRAVO mediation toolbox (BRAVO, 2014).

## Results

### Moving to an Active and Engaged Supported Living Environment Influences AMS

AMT increased over time, estimate [95% confidence interval] was 0.793 [0.276–1.309]; the negative interaction effect confirmed less increase in AMT with time for control participants than for residents −0.497 [−0.900 to −0.095]. This is illustrated in Appendix 4 in Supplementary Material. Hypothesis 1 was thus confirmed.

### Association of AMS With Number of Activities

**Figure 1:**
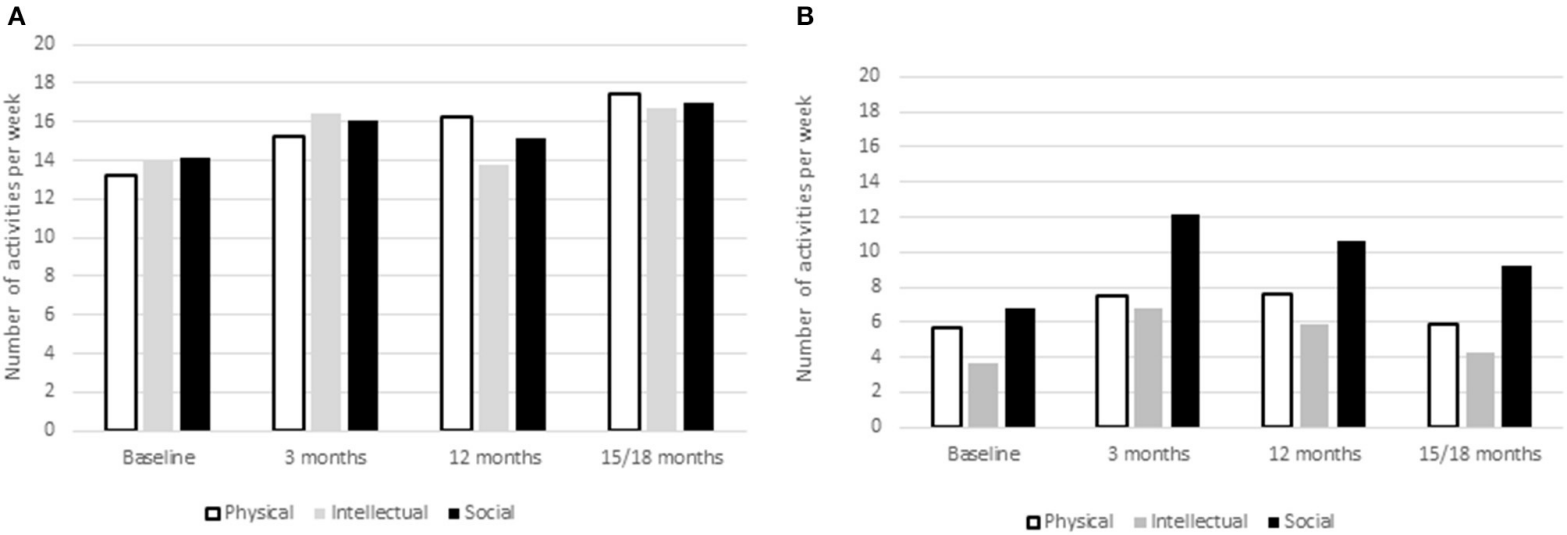
Number of physical, intellectual, and social activities per week from Diary Data for residents. Diary recorded activity over repeated measures **(A)** controls and **(B)** residents.

The above analysis demonstrated that the change in AMT varied by group, supporting impact of the generally active environment. Descriptive diary data demonstrate an increase in activities over the first 3 months but then a drop again, such that only the category of social activity demonstrated sustained increase (Figure 1). Importantly, at baseline there were five diary keepers reporting no engagement at all and although throughout the study there were still small numbers of people reporting no physical or intellectual activity, after baseline everyone reported at least one social activity per week. Correlation (across time periods) between total number of diary activities and AMT (residents data only), was significant: *r*(223) = 0.21, *p* < 0.01. In order to examine whether the potential effect of mood could impact diary activities, correlations between depression score and diary activity measures were conducted. Only the relationship between depression and social activities was significant, *r*(199) = −0.186, *p* < 0.01.

In the growth model, total number of diary activities did not have significant impact on observed increase in AMT. Specific models were computed for the three different types of activities, physical, social, and intellectual. Intellectual activities had a marginally significant (*p* = 0.07) impact on AMT overall: Estimate = 0.084, [−0.007 to 0.17], but did not interact with time. Social activity did not significantly impact AMT or interact with effect of time. However, given the above relationship between social activities and depression, when the analysis was repeated controlling for depression, social activity did significantly impact AMT, Estimate = 0.117, [0.041–0.193] but did not impact the time effect. Physical activity showed a significant impact on AMT: Estimate = 0.111, [0.013–0.209], but again there was no interaction with time. Thus, Hypothesis 2 was mostly confirmed. In order to further check whether activities were impacting types and period of memories, 100 recorded memories were examined for each group, taken from the last two assessment instances. Participants were not asked to date their memories although 86% of memories had a clear time period such as “last week” or “when I was at school.” There were no group differences in time period from which participants recalled memories although there were more recent than early memories overall (*F*_(3,54)_ = 2.98, *p* < 0.05). Residents did include some memories from their retirement village activities, but family, holiday and working life memories dominated for all participants.

### AMS as an Intermediary Between Cognitive Function and Perceived Health, Social Functioning, and Independence Outcomes

The role of AMT in mediating relationships between ACE-R and fluency (as a measure of executive function) on the one hand, and independence measures (IADLs and FLP: total FLP, communication, recreation, and social FLP) and perceived health (as a measure of well-being) on the other was examined at each time point. Table 1 summarises combinations in which AMT was a significant mediator and plots (a) and (b) in Appendix 5 in Supplementary Material indicate effect size. AMT positively mediated impact of fluency on perceived health: *95% CIs*: Baseline [0.039–0.116]; 3 months [0.027–0.128], 12 months [0.016–0.108], and 18 months [0.002–0.105]. AMT also mediated impact of general cognition on functional limitations, e.g., in the social category: at 12 months [−0.303 to −0.015] and 18 months, [−0.305 to −0.034], where negative mediation implies reduction of limitations. Thus, the effect anticipated in Hypothesis 3 is specific to executive function where perceived health is the outcome, but specific to general cognition where functional limitations are the outcome (at specific time points). In all cases, impact of AMT was as a positive mediator, increasing perceived health or reducing functional limitations.

**Table 1:** Autobiographical memory test (AMT) scores as a mediator between underlying cognitive functions or depression on the one hand, and outcome independence, perceived health, and functional limitations on the other.

**Relationship: AMT as a mediator between**	**Baseline**	**3 months**	**12 months**	**18 months**
ACE-R and perceived health	√	x	x	x
ACE-R and IADL	x	x	x	x
ACE-Rand functional limitations (Social)	x	x	√	√
ACE-R and functional limitations (Recreation)	x	x	√	x
ACE-R and functional limitations (communication)	x	x	x	x
ACE-R and total functional limitations	x	x	√	x
Depression and Perceived health	√	√	√	√
Depression and IADL	√	√	√	√
Depression and functional limitations (Social)	x	x	x	x
Depression and functional limitations (Recreation)	√	x	√	√
Depression and functional limitations (communication)	√	x	x	x
Depression and total functional limitations	√	√	√	√
Fluency and perceived health	√	√	√	√
Fluency and IADL	√	x	x	x
Fluency and functional limitations (Social)	x	x	x	x
Fluency and functional limitations (Recreational)	x	x	x	x
Fluency and functional limitations (communication)	x	x	x	x
Fluency and total functional limitations	x	√	x	x

*x = no significant mediation effect, √ = significant mediation effect*.

### AMS as an Intermediary Between Underlying Mood and Perceived Health, Social Functioning, and Independence Outcomes

The same mediation models were examined to assess impact of depression on outcome function (Table 1). AMT reliably mediated impact of depression level on outcome IADLs (*95% CIs*: Baseline [0.689–0.990]; 3 months [0.610–0.912]; 12 months [0.610–0.987]; 18 months [0.613–0.986]), on perceived health (Baseline [0.320–0.468]; 3 months [0.288–0.435]; 12 months [0.270–0.452]; 18 months [0.269–0.463]), on recreational limitations except for at 3 months (Baseline [0.949–2.695]; 12 months [0.332–1.926]; 18 months [0.405–2.459]), and on total functional limitations: (Baseline [5.478–16.969]; 3 months [7.029–18.079]; 12 months [7.740–18.779]; 18 months [6.674–20.987]), confirming hypotheses. Plot (c) Appendix 5 in Supplementary Material indicates effect size.

### Impact of AMS on the Mediating Effect of Depression Between Cognitive Functioning and Outcome Independence, and Functional Limitations

**Figure 2:**
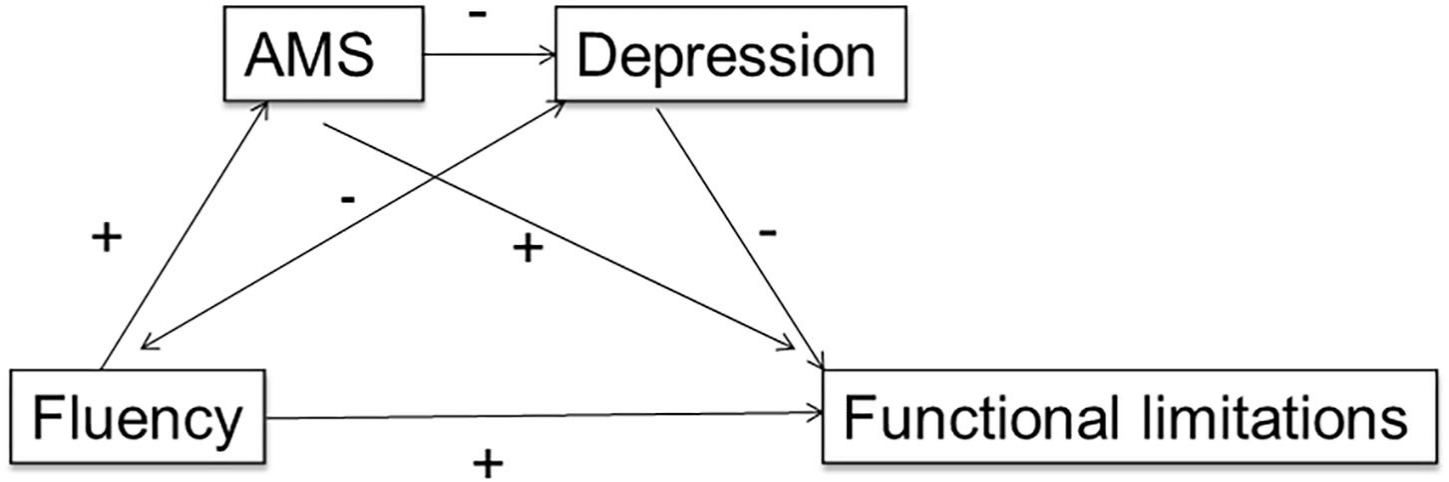
Multiple mediation model showing the forward effect of autobiographical memory specificity (AMS) on depression, as mediators between a measure of executive function (verbal fluency) and outcome functional limitations.

Given relationships between the three sets of variables, cognition, depression, and functional independence, the apparent intermediary role of our socio-emotional measure of cognition (AMT), and clear mediating impact of this on the effect of depression, we examined a multiple mediation model of the impact of both AMS and depression. In the relationship between fluency and total functional limitations (Figure 2), depression had the expected mediation effect, higher depressive symptoms increasing functional limitation (*95% CIs*: Baseline [10.881–17.449], 3 months [12.573–20.292], 12 months [10.350–16.307], 18 months [9.801–16.388]), and better AMS performance had a positive direct mediation effect, significant for baseline [0.354–15.981], and 3 month data [8.708–24.430] only, showing that mediation of AMS reduces over time. However, AMS significantly *reduced* the mediation effect of depression throughout the timeline (Baseline [0.350–0.492]; 3 months [0.353–0.520]; 12 months [0.300–0.457]; 18 months [0.300–0.457]). Effect size is illustrated by the bounds of the confidence intervals. Better autobiographical memory specificity is diminishing the mediation effect of depression on functional limitations.

**Figure 3:**
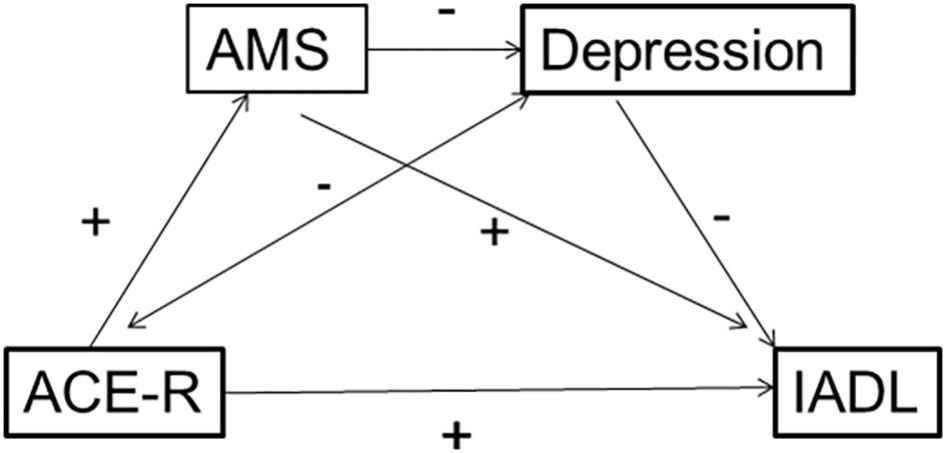
Multiple mediation model showing the forward effect of autobiographical memory specificity (AMS) on depression, as mediators between a measure of cognitive function (ACE-R) and outcome Instrumental Activities of Daily Living (IADL), in a context where the mediation effect of AMS between ACE-R and IADL itself is not significant.

The second model (Figure 3) examined the relationship between general cognition (ACE-R), and outcome IADL. Depression had a negative mediation effect on IADL except at 18 months (Baseline [−0.007 to −0.0007], 3 months [−0.011 to −0.004], 12 months [−0.007 to −0.0002], 18 months [−0.005 to 0.004]), but AMS did not. Nevertheless, AMS still reduced the mediation effect of depression (Baseline [0.377–0.516]; 3 months [0.366–0.531]; 12 months [0.301–0.461]; 18 months [0.293–0.456]). In both cases, the effect does not work the other way around; depression does not influence the mediating effect of AMS.

### Longitudinal Influence of AMS on Functional Limitations, Independence, and Well-Being

The influence of AMS on change over time for measures of social functioning and independence outcomes was assessed for residents only, given their AMS improvement, using a growth model as above, with the AMS^*^time interaction being the effect of interest.

For IADL there was an effect of time (estimate [*95% CI*]: 0.239 [0.038–0.439]), of AMS (0.145 [0.053–0.238]) and an interaction (−0.043 [−0.075 to −0.011]), illustrating that effect of AMS on IADL reduced over time. For FLP total there was a marginal (*p* = 0.064) effect of time (35.69 [−2.16–73.54]), but no effect of AMS or interaction.

For FLP communication, there was a marginally significant time^*^AMS interaction (*p* = 0.06, −0.846 [−1.72 to 0.03]), again illustrating that effect of AMS reduced over time. For FLP recreation and social, and depression, there were no longitudinal impacts of AMS. For perceived health, there was an effect of time (0.210 [0.051–0.368]), AMS (0.111 [0.043–0.179]) and an AMS^*^time interaction (−0.028 [−0.053 to −0.002]): perceived health improved over time and was positively impacted by AMS, but this impact reduced over time.

Change variables were computed, subtracting Baseline from 18 month data. Change in AMS was correlated with change in total FLP, *r*(93) = 0.26, *p* < 0.05, but not with change in IADL.

## Discussion

The study confirmed that autobiographical memory specificity (AMS) as measured by the AMT improved over time for people moving into the active supported living environment, with less improvement/more decline for the control group who were not making any changes, confirming the effect of the new environment. Two main factors explain this effect. The first is that the generally socially accessible environment is having an impact on this functional measure of cognition, with previous evidence showing an effect of increasing social networks on cognition and well-being (Bennett et al., 2014). Our diary data demonstrated a reduction in the numbers of people reporting no social engagement and parallel qualitative studies with the same sample provide convergence of evidence in that participants reported an increase in “having company” and reduction in loneliness (Holland et al., 2015, 2019). Other publications support this, reporting that people living in extra care type establishments reported lower loneliness than the background population living in private accommodation (Beach, 2015), and our 2019 analysis showing that residents in these retirement villages were less likely to be lonely on average than older people in the general English population, and loneliness also showing an association with AMS (Holland et al., 2019). The second factor is the social situation in which new residents find themselves, especially to start with. They were making new acquaintances and friends, introducing who they are to people who do not know them, solving social problems such as negotiating their role in a new club or activity, while dealing with the trauma of moving home sometimes in the context of changing health care needs or bereavements. Autobiographical memory specificity plays a significant role in all of these activities and so receives considerable “practice” in the early months of adjustment. Participants did not date their memories, but a sample suggested that recent events were more strongly represented than earlier events for both groups. This contrasts with earlier work based in traditional (less active) care homes whereby residents' recall was predominantly of earlier memories (Holland and Rabbitt, 1991), with authors also relating this to practice via reminiscence. If this concept is correct, one may expect that both improvement in AMS and its influence on factors such as perceived health or functional limitations may have more of an impact in the earlier months of settling in. This is indeed the finding, with the longitudinal models showing reduction of the significant influence of AMS over time (the AMS × time interactions) on IADL, perceived health and, marginally, on functional limitations in communication. This study therefore confirms AMS as a functional aspect of socio-emotional cognition that has direct impact on everyday functioning and coping in challenging social and emotional situations. Factors that enable improvement of AMS, such as increased availability of social interaction, clearly have an impact on measures of independence. These relationships go both ways; those with poorer AMS showed reduced perceived health and functional independence over the period. The relationships between diary reported physical and social activities and AMS support this, and future work with a greater number of diary keepers, or other methods of assessing amount of physical activity could usefully distil direct physical activity impacts on outcomes such as functional independence from mediated impact of AMS.

The suggestion that AMS may be an important functional intermediary between underlying cognition or depression, and outcome functional and well-being indicators, was supported for executive function as a predictor of perceived health, with AMS mediation of the predictive relationship persisting across time. Self-perceived health is a crucial variable to explore in a group potentially at risk of increasing needs and frailty; it encompasses health status, elements of health behaviours, psychological state and emotional well-being (Jylha, 2009). Previous evidence demonstrates reliable association to objectively measured health, and prospectively to healthcare utilisation, morbidity and mortality (e.g., Lima-Costa et al., 2012). Given such relationships, improvement in perceived health could have far reaching positive consequences on survival and care needs and costs. This reliable impact of both underlying executive function and mediation of AMS gives strong support for rehabilitative efforts in both functions.

AMS mediation of the relationship of general cognition with social functional limitations supported the proposal that ability to recall specific autobiographical experiences impacts ability to socially engage. Given evidence of a relationship between social networks and cognitive impairment outcomes (Bennett et al., 2014), autobiographical memory is proposed as a malleable function that could have an important impact on such outcomes.

The hypotheses raised in the introduction suggested that AMS may be an important functional intermediary between underlying variables such as overall cognitive function, executive function, and depression, and outcome functional independence. This was supported at baseline for executive function as a predictor of IADL (but not for general cognition), but this relationship did not persist across time. Nevertheless, AMS did mediate the relationship of general cognition with outcome functional limitations at some time points, particularly at 12 months.

Multiple mediation models confirmed the role of AMS on the effect of depression on everyday functioning and this was reliable across time. AMS had a forward effect on depression as a mediator between cognition and outcome IADL and functional limitations, even where it was not a mediator on its own. This is further support for a focus on AMS in rehabilitative strategies for older adults and confirms the central role of the function of autobiographical memory in the effect of depression on everyday functioning. That is, AMS has an influence on the manner in which depression mediates cognition and function, further support for a focus on AMS in rehabilitative strategies for older adults, with Leahy et al. (2018a) demonstrating an impact of AMS training on social problem solving and functional limitations in older adults (and so functional independence), and for strategies where AMS rehabilitation is incorporated into treatment for clinical levels of depression (Raes et al., 2009).

### Limitations

These analyses have not considered issues such as mobility or sensory impairments that may also be having an effect on outcome functions and engagement, and have not distinguished people with clinical levels of depression or significant cognitive impairment. Instead, we have used measures of depression and cognition as continua (within the bounds of the inclusion criterion of having mental capacity to consent). In addition, there is no measure of social interaction or diary data from before the residents moved in, but self-reports in the parallel qualitative data do provide evidence for an increase in social interaction and opportunities for companionship (Holland et al., 2015). Finally, we do not have a measure of quality of life for these periods, which would be useful to add in future analyses.

## Conclusions

This study has demonstrated that an active and socially accessible environment can have a significant influence on autobiographical memory specificity on older adults. It has also shown that this socio-emotional, functional, aspect of memory has a significant role in mediating the effect of underlying cognitive capabilities and mood/depression on outcome independent functioning and perceived health, all crucial components of quality of life with implications for care needs and costs. It is proposed that factors or programmes that improve autobiographical memory specificity could have very significant consequences on outcomes for older adults.

## Author's Note

All authors were affiliated to Aston Research Centre for Healthy Ageing, Aston University, Birmingham, UK, at the time of the initial data collection.

## Data Availability Statement

The raw anonymized data supporting the conclusions of this article can be made available by the authors, without undue reservation.

## Ethics Statement

The studies involving human participants were reviewed and approved by Aston University Research Ethics Committee. The patients/participants provided their written informed consent to participate in this study.

## Author Contributions

The longitudinal project from which the data in this paper comes was managed throughout by CH, the Principal Investigator, who also wrote the original grant proposal. CH led on the rationale for this paper and wrote all drafts. AB made significant contribution to the analysis of the data, notably the mediation analyses, contributed to the reporting of these analyses, and critical revision of the final draft for intellectual content. RC led on the diary component of the study from conception and was involved in critically revising the final draft for intellectual content. DC led on the data management, contributed to data collection, and to critical revision of the paper for intellectual content. All authors contributed to the article and approved the submitted version.

## Conflict of Interest

The authors declare that the research was conducted in the absence of any commercial or financial relationships that could be construed as a potential conflict of interest.

## Footnotes

^1^Extra care housing, increasingly prevalent in the UK, has the aim of meeting “the housing, care and support needs of older people, while helping them to maintain their independence in their own private accommodation” (Darton et al., 2011, p. 77). In the 13 different locations included in this study, provided by the ExtraCare Charitable Trust, there are additional services such as a “well-being” advisor (a nurse) for health assessment and support, and specific support for residents living with dementia or mild cognitive impairment, and an approach in which active engagement includes volunteering and leading activities, rather than simple provision. They are largely modern, purpose-built or adapted environments with attention paid to accessibility and community meeting areas such as coffee shops and village “centres”.

^2^At later stages of the project the ACE-R was replaced by the equivalent ACE-III (Hsieh et al., 2013).

## References

[B1] Alea N. Bluck S. (2003). Why are you telling me that? A conceptual model of the social function of autobiographical memory. Memory 11, 165–178. 10.1080/741938207 12820829

[B2] Beach B. (2015). Village Life Independence, Loneliness, and Quality of Life in Retirement Villages with Extra Care. London: ILC-UK. Available online at: https://ilcuk.org.uk/village-life/ (accessed March 29, 2021).

[B3] Beaman A. Pushkar D. Etezadi S. Bye D. Conway M. (2007). Autobiographical memory specificity predicts problem-solving ability in old and young adults. Q. J. Exp. Psychol. 60, 1275–1288. 10.1080/17470210600943450 17676558

[B4] Bennett D. A. Arnold S. E. Valenzuela M. J. Brayne C. Schneider J. A. (2014). Cognitive and social lifestyle: links with neuropathology and cognition in late life. Acta Neuropathologica 127, 137–150. 10.1007/s00401-013-1226-2 24356982 PMC4054865

[B5] Biggs S. Bernard M. Kingston P. Nettleton H. (2000). Lifestyles of belief: narrative and culture in a retirement community. Ageing Soc. 20, 649–672. 10.1017/S0144686X01007930

[B6] BRAVO (2014). BRAVO: Bootstrap Regression Analysis of Voxelwise Observations (version 2.0) BRAVO Mediation - BRAVO Toolbox. Site visited on February 15, 2021.

[B7] da Silva J. Gonçalves-Pereira M. Xavier M. Mukaetova-Ladinska E. B. (2013). Affective disorders and risk of developing dementia: systematic review. BJP 202, 177–186. 10.1192/bjp.bp.111.101931 23457181

[B8] Dalgleish T. Williams J. M. G. Golden A. M. J. Perkins N. Barrett L. F. Barnard P. J.. (2007). Reduced specificity of autobiographical memory and depression: the role of executive control. J. Exp. Psychol. 136, 23–42. 10.1037/0096-3445.136.1.23 17324083 PMC2225543

[B9] Darton R. Bäumker T. Callaghan L. Netten A. (2011). Improving housing with care choices for older people: the PSSRU evaluation of extra care housing. Housing Care Support 14, 77–82. 10.1108/14608791111199741

[B10] Goddard L. Dritschel B. Burton A. (1997). Social problem solving and autobiographical memory in non-clinical depression. Br. J. Clin. Psychol. 36, 449–451. 10.1111/j.2044-8260.1997.tb01252.x 9309360

[B11] Hofer S. M. Sliwinski M. J. (2006). Design and analysis of longitudinal studies in aging, in Handbook of the Psychology of Aging, 6th Edn, eds Birren J. E. Schaie K. W. (San Diego, CA: Academic Press), 15–37. 10.1016/B9-78-012101-2/64950-0057

[B12] Holland C. A. Rabbitt P. M. (1991). Ageing memory: use versus impairment. Br. J. Psychol. 82, 29–38. 2029603 10.1111/j.2044-8295.1991.tb02380.x

[B13] Holland C. A. Boukouvalas A. Wallis S. Clarkesmith D. Cooke R. Liddell L.. (2017). Transition from community dwelling to retirement village in older adults: cognitive functioning and psychological health outcomes. Ageing Soc. 37, 1499–1526. 10.1017/S0144686X16000477

[B14] Holland C. A. Carter M. Cooke R. Leask G. Powell R. Shaw R.. (2015). Collaborative Research between Aston Research Centre for Healthy Ageing (ARCHA) and the ExtraCare Charitable Trust: The Final Report. Available online at: ExtraCare report 2015.pdf (accessed January 12, 2021).

[B15] Holland C. A. Garner I. O'Donnell J. Gwyther H. (2019). Integrated Homes, Care And Support: Measurable Outcomes For Healthy Ageing. Available online at: https://www.extracare.org.uk/media/1169231/full-report-final.pdf (Extracare.Org.Uk) (accessed January 10, 2021).

[B16] Holland C. A. Rabbitt P. M. (1990). Autobiographical and text recall in the elderly: an investigation of a processing resource deficit. Q. J. Exp. Psychol. 42a, 441–470. 10.1080/14640749008401232 2236630

[B17] Holland C. A. Ridout N. Walford E. Geraghty J. (2012). Executive function and emotional focus in autobiographical memory specificity in older adults. Memory 20, 779–793. 10.1080/09658211.2012.703210 22873516

[B18] Hsieh S. Schubert S. Hoon C. Mioshi E. Hodges J. R. (2013). Validation of the Addenbrooke's cognitive examination III in frontotemporal dementia and Alzheimer's disease. Dement. Geriatr. Cogn. Disord. 36, 242–250. 10.1159/000351671 23949210

[B19] Idler E. L. Benyamini Y. (1997). Self-rated health and mortality: a review of twenty-seven community studies. J. Health Soc. Behav. 38, 21–37. 10.2307/2955359 9097506

[B20] Jenkins K. Pienta A. Horgas A. (2002). Activity and health related quality of life in continuing care retirement communities. Res. Aging 24, 124–149. 10.1177/0164027503024001008

[B21] Jorm A. F. (2001). History of depression as a risk factor for dementia: an updated review. Aust. N. Z. J. Psychiatry 35, 776–781. 10.1046/j.1440-1614.2001.00967.x 11990888

[B22] Jylha M. (2009). What is self-rated health and why does it predict mortality? Towards a unified conceptual model. Soc. Sci. Med. 69, 307–316. 10.1016/j.socscimed.2009.05.013 19520474

[B23] Lawton M. P. Brody E. M. (1969). Assessment of older people: self-maintaining and instrumental activities of daily living. Gerontologist 9, 179–186. 10.1093/geront/9.3_Part_1.179 5349366

[B24] Leahy F. Ridout N. Holland C. (2018b). Memory flexibility training for autobiographical memory as an intervention for maintaining social and mental well-being in older adults. Memory 26, 1310–1322. 10.1080/09658211.2018.1464582 29733760

[B25] Leahy F. Ridout N. Mushtaq F. Holland C. (2018a). Improving specific autobiographical memory in older adults: impacts on mood, social problem solving, and functional limitations. Aging Neuropsychol. Cogn. 25, 695–723. 10.1080/13825585.2017.1365815 28825508

[B26] Leontjevas R. L. Gerritsen D. Smalbrugge M. Teerenstra S. Vernooij-Dassen M. Koopmans R. (2013). A structural multidisciplinary approach to depression management in nursing-home residents: a multicentre, stepped-wedge cluster-randomised trial. Lancet 381, 2255–2264. 10.1016/S0140-6736(13)60590-5 23643110

[B27] Lima-Costa F. M. Steptoe A. Cesar C. C. De Oliveira C. Proietti F. A. Marmot M. (2012). The influence of socioeconomic status on the predictive power of self-rated health for 6-year mortality in English and Brazilian older adults: the ELSA and Bambui cohort studies, Ann. Epidemiol. 22, 644–648. 10.1016/j.annepidem.2012.06.101 22819435

[B28] Mioshi E. Dawson K. Mitchell J. Arnold R. Hodges J. R. (2006). The Addenbrooke's cognitive examination revised (ACE-R): a brief cognitive test battery for dementia screening. Int. J. Geriatr. Psychiatry 21, 1078–1085. 10.1002/gps.1610 16977673

[B29] Noone P. (2015). Addenbrooke's cognitive examination-III. Occup. Med. 65, 418–420. 10.1093/occmed/kqv041 26187808

[B30] Piolino P. Coste C. Martinelli P. Mace A.-L. Quinette P. Guillery-Gurard B.. (2010). Reduced specificity of autobiographical memory and aging: do the executive and feature binding functions of working memory have a role? Neuropsychologia 48, 429–440. 10.1016/j.neuropsychologia.2009.09.035 19804792

[B31] Piolino P. Desgranges B. Benali K. Eustache F. (2002). Episodic and semantic remote autobiographical memory in aging. Memory 10, 239–257. 10.1080/09658210143000353 12097209

[B32] Pollard B. Johnston M. (2001). Problems with the Sickness Impact Profile: a theoretically-based analysis and a proposal for a new method of implementation and scoring. Soc. Sci. Med. 52, 921–934. 10.1016/S0277-9536(00)00194-5 11234865

[B33] Preacher K. J. Hayes A. F. (2008). Asymptotic and resampling strategies for assessing and comparing indirect effects in multiple mediator models. Behav. Res. Methods 40, 879–891. 10.3758/BRM.40.3.879 18697684

[B34] Raes F. Hermans D. Williams J. M. G. Eelen P. (2006). Reduced autobiographical memory specificity and affect regulation. Cogn. Emotion 20, 402–429. 10.1080/02699930500341003 26529213

[B35] Raes F. Williams J. M. G. Hermans D. (2009). Reducing cognitive vulnerability to depression: a preliminary investigation of Memory Specificity Training (MeST) in inpatients with depressive symptomatology. J. Behav. Ther. Exp. Psychiatry 40, 24–38. 10.1016/j.jbtep.2008.03.001 18407245

[B36] Ros L. Latorre J. M. Serrano J. P. (2010). Working memory capacity and overgeneral autobiographical memory in young and older adults. Aging Neuropsychol. Cogn. 17, 89–107. 10.1080/13825580903042650 19626477

[B37] Singer J. D. Willett J. B. (2003). Applied Longitudinal Data Analysis: Modeling Change and Event Occurrence. Oxford: Oxford University Press.

[B38] Williams J. M. Barnhofer T. Crane C. Hermans D. Raes F. Watkins E.. (2007). Autobiographical memory specificity and emotional disorder. Psychol. Bull. 133, 122–148. 10.1037/0033-2909.133.1.122 17201573 PMC2834574

[B39] Williams J. M. Broadbent K. (1986). Autobiographical memory in suicide attempters. J. Abnorm. Psychol. 95, 144–149. 10.1037/0021-843X.95.2.144 3711438

[B40] Zigmond A. S. Snaith R. P. (1983). The hospital anxiety and depression scale. Acta Psychiatr. Scand. 67, 361–370. 10.1111/j.1600-0447.1983.tb09716.x 6880820

