# Supplementary material for: Specific Autobiographical Recall Mediates Impact of Cognition and Depression on Independence Function and Well-Being in Older Adults
Source: Front Psychol. 2021 Apr 20;12:652600. doi: 10.3389/fpsyg.2021.652600 (PMC8095394; doi:10.3389/fpsyg.2021.652600)

Supplementary Material

Appendix 1:

Numbers added at each stage and per cent attrition across the 18 month study

Group	Reason for attrition	Baseline	% attrition	3 months	% attrition	12 months	% attrition	18 months	Total attrition
**Residents**		162	17%	134	17%	111	12.6%	97	40%
	Died	3		4		5			7.4%
	Too ill	4							
	other	21							
Added		162		10		10		1	
**Total**		162		144		127		108	
Dairy sample		57		50		58		34	
**Control**		31	3%	30	16.7%	25	12%	22	29%
	Died	0		0		0			
	Too Ill								
	other	1							
Added		31		2		6		0	
**Total**		31		32		31		29	
Diary sample		22		19		17		12	
**Total of original at each time point**		193	15%	164	17%	136	12.5%	119	38.3%
**Total final sample**		193		176		159		137	29.01%
**Total Diary Sample at each time point**		79		69		75		46	

Appendix 2

Autobiographical memory test instructions

- I am interested in your memory for events that have happened in your life. I am going to read you some words. For each word I want you to think of an event that happened to you which the word reminds you of. The event could have happened recently (yesterday/last week) or a long time ago. It might be an important event or a trivial event.
- The memory you recall should be a specific event. So if I said the word 'good' – it would not be OK to say 'I always enjoy a good party', because it does not mention a specific event
- It would be ok to say 'I had a good time at Jane's party last month' because that is a specific event
- It would not be a specific episodic memory if you said "I graduated on 7th July 2012"

Try to retrieve a different memory for each word.

- When doing AMT, use the two different lists of 10 words randomly.

List 1	List 2
Bored	Scared
Clever	Relaxed
Nervous	Confused
Safe	Love
Clumsy	Lost
Happy	Funny
Disappointed	Embarrassed
Calm	Impressed
Sad	Irritable
Proud	Determined

AMT item scoring:
4. specific to a particular day
3. extended - lasted more than 1 day – I felt calm last summer
2. categorical – a class of events – I feel calm when I watch a film.
1. semantic associate – not a memory, but semantic reasoning – I would feel calm when at the sea.
0. can't think of anything

Overall score in SPSS = the number of 4s. The range is 0 to 10.

Appendix 3:

Participant Diary

How to fill in your diary

•Thank you for agreeing to help us with our study. Here are some points to bear in mind when filling in your diary. Remember that this is **YOUR** diary and filling it out can help you to keep a record of all of your week's activities:

•Your diary should be filled out every day during the period the researcher will tell you.

•You will have an opportunity to discuss any of your entries with the researcher.

•Please don't worry about spelling, grammar or 'best' handwriting but try to write as clearly as you can, using a pen.

•If you find that you have missed out several days, please do not give up the whole week's diary. Just start again on the next day you are able to fill it in, and leave the other pages blank.

For each entry you need to write down:
1. The **name of the activity** (e.g. Chess club, swimming, lunch with a friend)
2. **Your role** (i.e. Did you go along and take part? Go along and watch? Or were you organising the event?).
3. The **type of activity** (e.g. intellectual, social or physical)
Intellectual (things that exercise your brain – e.g. a crossword puzzle, a game of cards)
Social (meeting with people, e.g. having a good chat on the phone or meeting with your friends or family)
Physical (some exercise – e.g. a walk, a dance class, going to the gym)
4. Also score **how much you enjoyed the activity** on a scale of 1-5 (1= i did not enjoy the activity at all, 5= I enjoyed the activity a lot)
By understanding how and when people engage in physical and social activity, we can see whether this impacts on people's health outcomes. If you have any concerns or queries about completing the diary we will be happy to discuss these with you.

Best wishes from
………………………………

**Activities** Week beginning (date) __/__/__	**Name of activity**	**Your role**	**Type of activity (tick box/es)**		**Enjoyment Score (1-5)**
			Physical		
			Intellectual		
			Social		
			Physical		
			Intellectual		
			Social		
			Physical		
			Intellectual		
			Social		
			Physical		
			Intellectual		
			Social		

Diary category examples, from coding

Physical:

Sport/leisure/exercise – bowls, golf, gym, swimming, fitness classes, etc

Walking – to and from places, clubs (leisure walking) etc

Gardening – trimming, mowing, planting,

Housework – any chores, decorating, house related tasks. Includes assembly of furniture

Shopping- grocery – food shopping, butchers, veg markets etc. Any food specified shopping

Shopping – leisure – clothes, stalls, Christmas shopping, any non specified shopping activity

Intellectual:

Correspondence – telephone, letters, writing (e.g. to solicitor or company)

Religious – communion, services, activities, lectures, sermons, etc

Puzzles – crossword, sudoku

Hobby/club/meeting – AGM, any other meeting, hobbies e.g. craft, sewing, knitting, bridge, etc

Current affairs, audio-visual – TV, radio, reading paper, any other reading, watching films etc

Computer work – PC games, emails etc

Admin work – utilities, bills, sorting out, arranging events/outings

Social:

Friends/visitors – meeting or hosting, chatting with other residents, social interaction with others

Social (coffee, lunch etc) – coffee, tea, lunch, dinner, meets with others/friends casual.

Social (other) – fêtes, social events, theatre, dinner out, concerts, family gatherings for occasions.

Relatives – seeing/meeting/contact with family members in any context

Celebrations – birthdays, anniversaries, etc

Appendix 4

Details of Autobiographical Memory Recall over Time

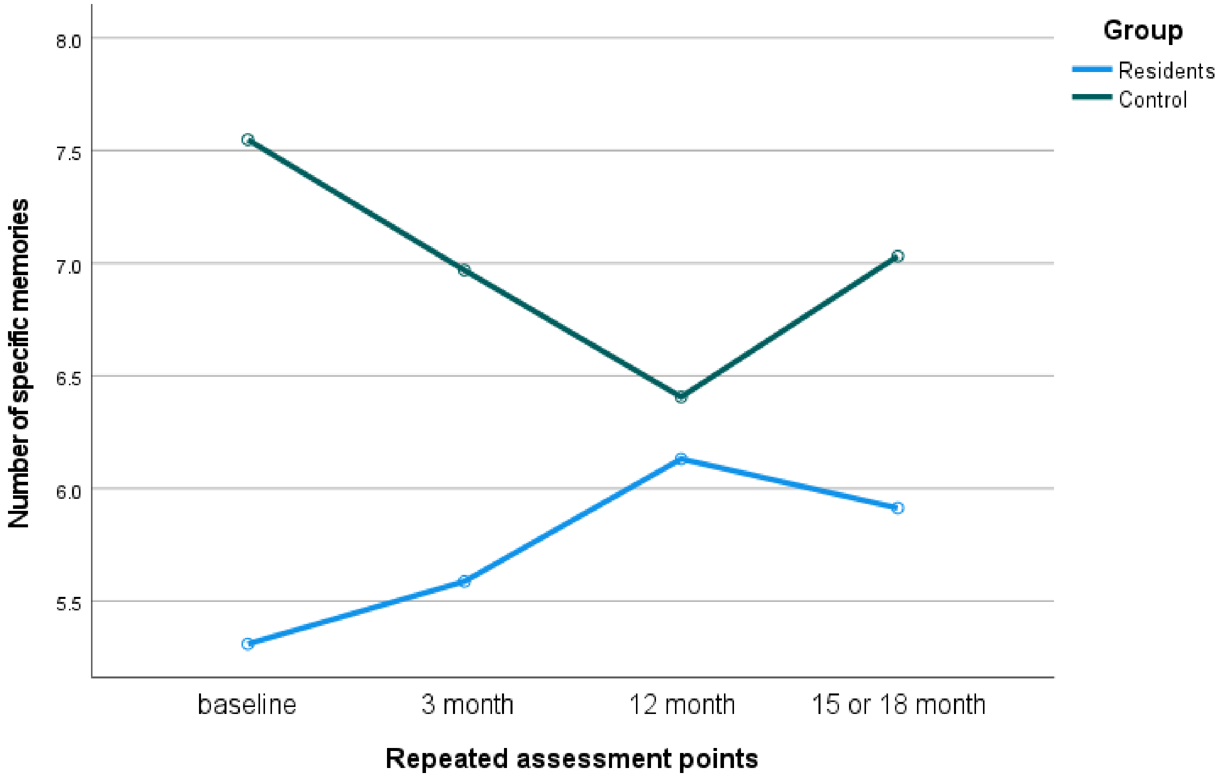

Appendix 5

Plots illustrating effect size for mediation analyses

In the plots below, we show the average mediation effect size of an input on different outputs. Due to the linearity of the mediation model this has a very simple interpretation: for a fixed value of the mediator, if we change the input by 1 unit, the response will change (on average) by the mean of the product of the linear coefficients. This is depicted by the colour bar in the plots. A negative effect suggests the mediated input has a dampening effect, i.e. increasing the input by 1 unit will reduce the response by the effect size shown.

We also show a 95% confidence interval calculated using the bootstrap approach of Preacher and Hayes (2008). If the confidence interval includes the zero value, the effect cannot be found significant.

(a) AMS as an Intermediary between Cognitive Function (ACE-R) and Perceived Health, Social Functioning and Independence Outcomes (IADL and FLP)

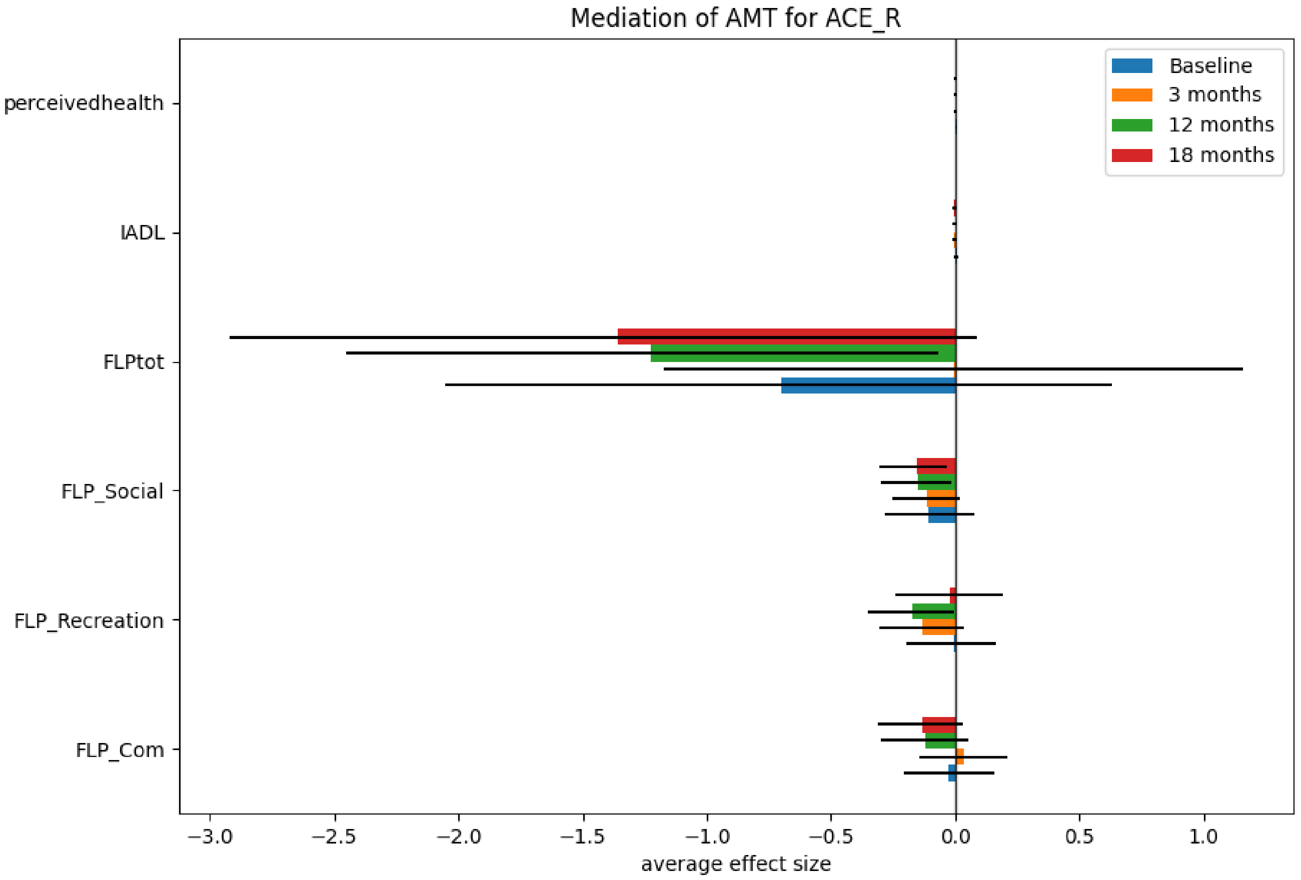

(b) AMS as an Intermediary between Executive Function (Fluency) and Perceived Health, Social Functioning and Independence Outcomes (IADL and FLP)

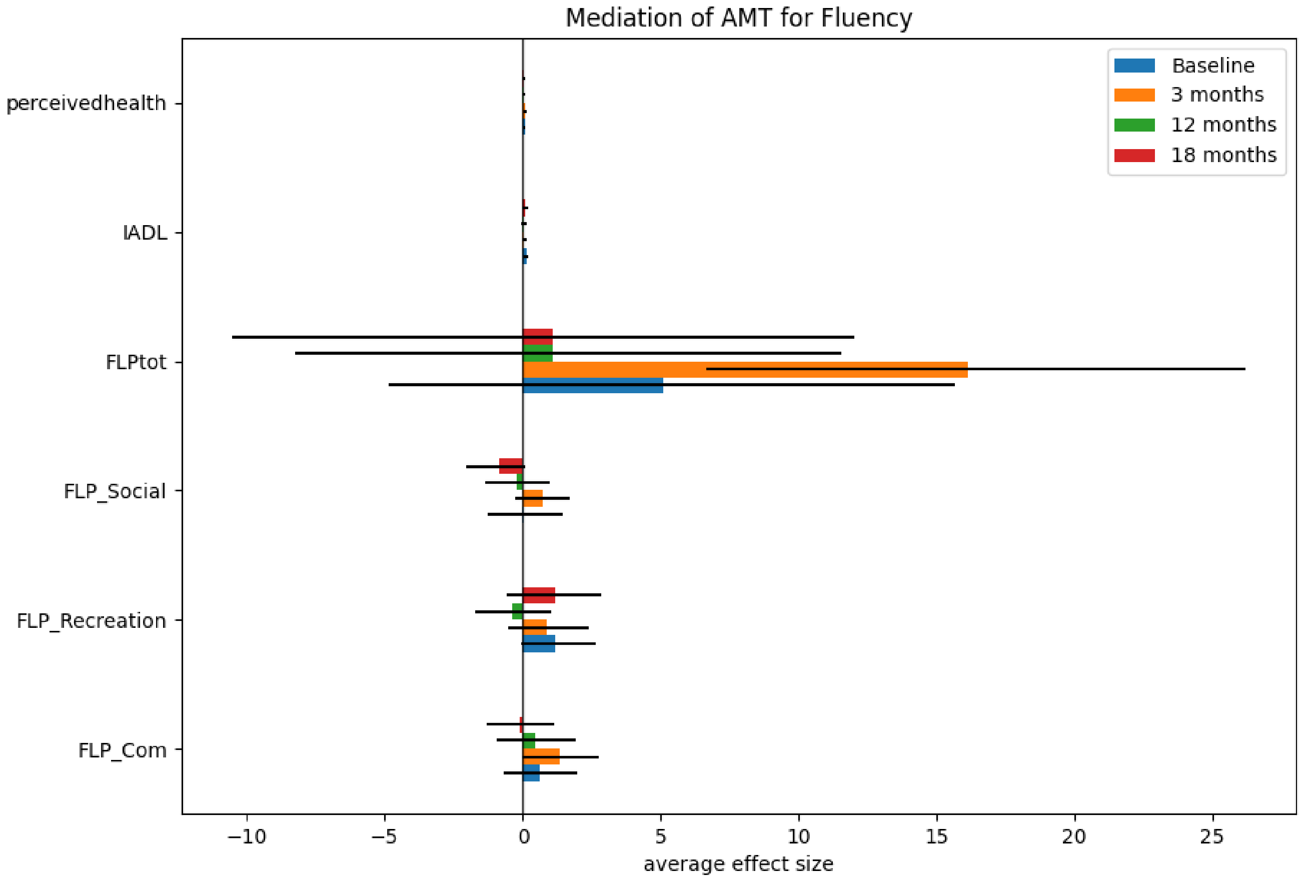

c) AMS as an Intermediary between Underlying Mood (HADS-D) and Perceived Health, Social Functioning

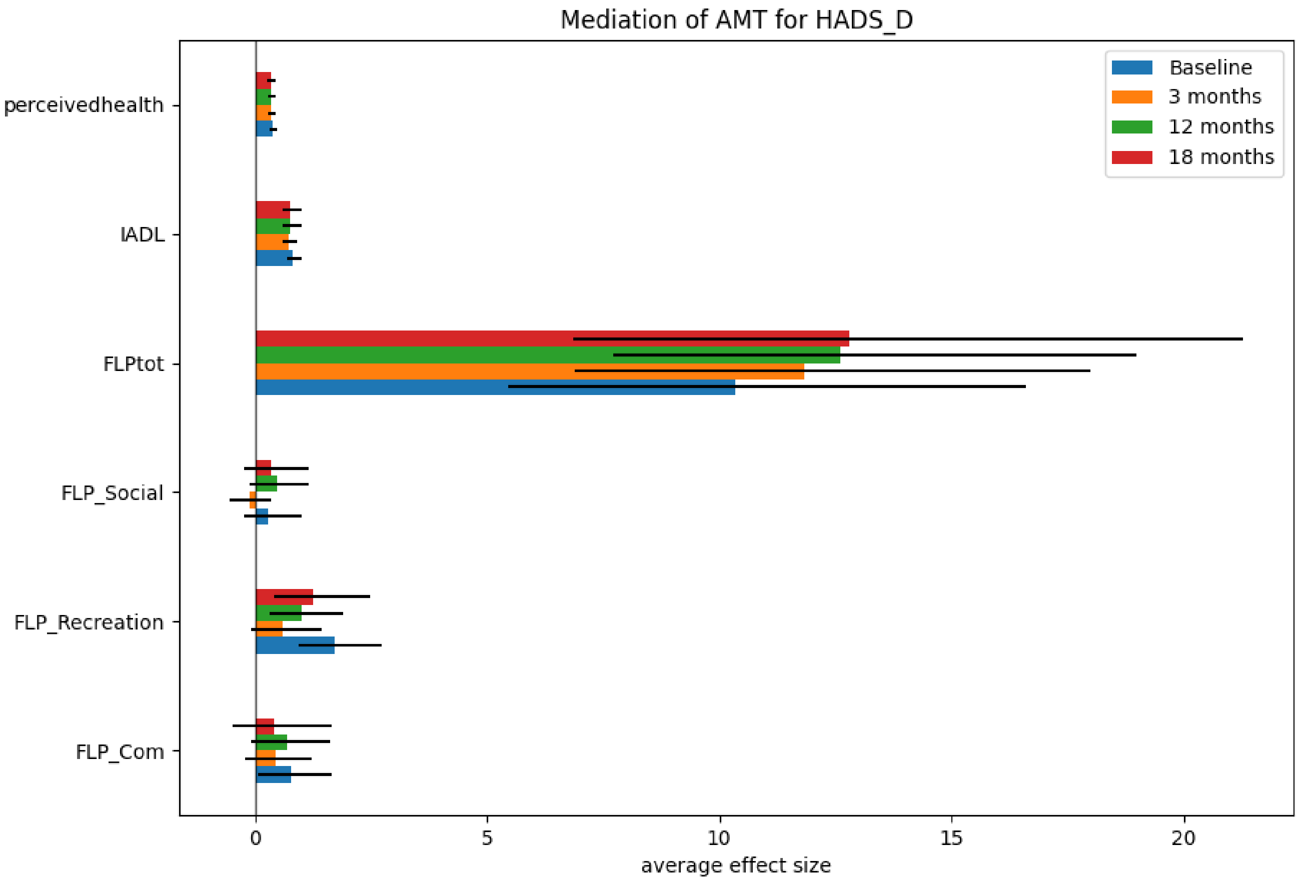

Supplement: Supplementary file 1 [file Data_Sheet_1.PDF]
